# An Extraordinary Case of Autoimmune Polyendocrinopathy-Candidiasis-Ectodermal Dystrophy (APECED) Syndrome Misdiagnosed as Juvenile Idiopathic Arthritis on Admission

**DOI:** 10.1155/2023/2363760

**Published:** 2023-04-25

**Authors:** Gulcin Aytac, Burcu Guven, Ilyas Aydin, Ezgi Topyildiz, Ayca Aykut, Asude Durmaz, Neslihan Edeer Karaca, Guzide Aksu, Necil Kutukculer

**Affiliations:** ^1^Ege University Faculty of Medicine, Department of Pediatric Rheumatology, Izmir, Turkey; ^2^Ege University Faculty of Medicine, Department of Pediatric Immunology, Izmir, Turkey; ^3^Ege University Faculty of Medicine, Medical Genetics, Izmir, Turkey

## Abstract

**Background:**

APECED is a syndrome characterized by autoimmune polyendocrinopathy, candidiasis, and ectodermal dystrophy. The most observed clinical findings are chronic mucocutaneous candidiasis, hypoparathyroidism, and autoimmune adrenal insufficiency. *Case Presentation*. A three-year-old male patient was admitted with classical signs of juvenile idiopathic arthritis and treated with nonsteroidal anti-inflammatory drugs. During follow-up, signs of autoimmunity, candidiasis, nail dystrophy, and onychomycosis were observed. The parents were consanguineous, and targeted next-generation sequencing was performed. A homozygous mutation in the AIRE gene SAND domain (c.769C > T, p.Arg257Ter) was detected, and the patient was diagnosed with APECED syndrome.

**Conclusion:**

Inflammatory arthritis is rarely described in association with APECED and is often misdiagnosed as juvenile idiopathic arthritis. In APECED cases, nonclassical symptoms such as arthritis may occur before developing classical symptoms and considering the diagnosis of APECED in patients with CMC and arthritis is useful for early diagnosis before development of complications and management of disease.

## 1. Background

APECED (autoimmune polyendocrinopathy-candidiasis-ectodermal dystrophy) is a syndrome characterized by multiple signs of autoimmunity [1]. Autoimmune polyglandular syndrome type 1 (APS-1) is the other name of APECED syndrome. It is a rare inherited disease that affects multiple organs and is characterized by three main symptoms caused by mutations in the autoimmune regulatory gene (*AIRE*) [2]. The total number of APECED patients worldwide is estimated to be approximately 1000 individuals [2]. Individuals affected as a result of *AIRE* gene mutation show endocrine and autoimmune manifestations characterized by autoantibody production. The classic triad of symptoms is chronic mucocutaneous candidiasis (CMC), hypoparathyroidism, and autoimmune adrenal insufficiency [1]. A few more organ-specific manifestations occur, including premature ovarian failure, autoimmune thyroid disease, type 1 diabetes, autoimmune gastritis and enteropathy, autoimmune hepatitis, alopecia, vitiligo, and keratitis [3]. More than 80–90% of APECED cases have chronic mucocutaneous candidiasis as the first symptom [4].

We present a case presenting with a nonclassical symptom of APECED, arthritis, and misdiagnosed as systemic juvenile idiopathic on admission who lately developed signs of autoimmunity and candidiasis. Our aim is to emphasize to think about possible APECED diagnosis when the clinicians encounter arthritis, rash, fever, and history of moniliasis.

## 2. Case Presentation

A three-year-old male patient was admitted with complaints of pain and swelling in both ankles and wrists and rash with fever for more than 6 weeks. In addition to these clinical findings, he was referred to our hospital for the evaluation of increased acute phase reactants and examination of splenomegaly and abdominal distension. In the physical examination, the patient had fever and significant swelling especially in the right knee.

The patient was the second child of consanguineous parents. There was a third-degree cousin marriage without any other parental comorbidities. The other two children of the family were doing well and did not have any health problems.

A preliminary diagnosis of systemic juvenile idiopathic arthritis (JIA) was considered because of complaints of fever, splenomegaly, rash, and arthritis lasting more than 10 days. Laboratory findings revealed an increase in acute phase reactants as C-reactive protein (CRP): 13.4 mg/dl (normal: 0–0.5), ferritin: 143.5 ng/ml (normal: 7–140), erythrocyte sedimentation rate (ESR): 26 mm/h (normal: <15 mm/h), and serum amyloid A (SAA): 209 mg/l (normal: <6.4). Hypergammaglobulinemia (IgG : 3810 mg/dl, IgA : 165 mg/dl, and IgM : 161 mg/dl) and positive antinuclear antibody (ANA) (1/160 cytoplasmic) were detected. Anti-double-stranded DNA antibody (Anti-DsDNA) was negative. His antistreptolysin O titer (ASO), rheumatoid factor (RF), and antineutrophil cytoplasmic antibody tests were all negative. PPD (purified protein derivative) intradermal test for tuberculosis examination resulted as anergic. Brucellosis and toxoplasmosis laboratory tests were both negative. Ophthalmologic examination for uveitis was found to be normal.

No pathological findings were detected in the chest X-ray. Since the patient had splenomegaly, bone marrow aspiration was performed for possible malignancies and macrophage activation syndrome. In the bone marrow examination, no signs of hemophagocytosis, atypical cells, and blasts were found while a few hypersegmented neutrophils were observed. Treatment of nonsteroidal anti-inflammatory drugs (NSAIDs) was started.

Arthritis, fever, and rash began to recover after two weeks of treatment. However, moniliasis was observed on the oral mucous membranes and tongue in the oropharyngeal examination. Additionally, nail dystrophy and onychomycosis in the right thumb were observed. Nail dystrophy was secondary to onychomycosis and occurred after 4-5 months after onychomycosis. The lymphocyte subset analysis by flow cytometry and T/B cell proliferation tests resulted normal. However, without genetic analysis, we could not exclude innate immune system deficiency, and intravenous immunoglobulin (IVIG) treatment (in a dose of 0.5 gm/kg, once in four weeks) was started as it was used both in systemic juvenile idiopathic arthritis and in innate immune deficiencies.

**Figure 1:**
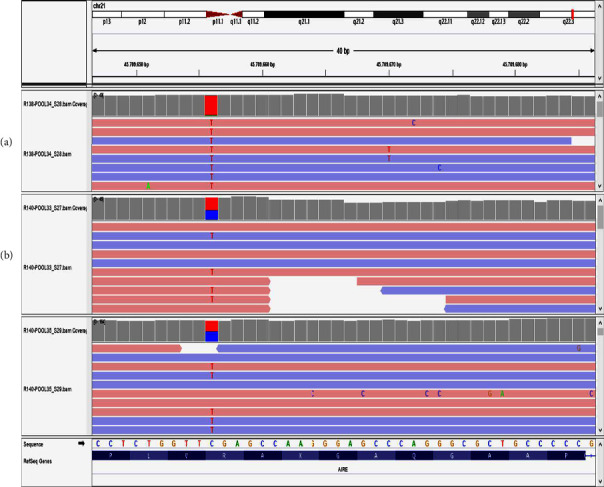
(a) Patient's AIRE gene sequence analysis images: homozygous c.769C > T (p.Arg257Ter) mutation in exon 6. (b) His parents' heterozygous c.769C > T (p.Arg257Ter) mutation in exon 6.

Then, genetic examination could be performed to exclude primary immunodeficiency in this case with chronic onychomycosis. STAT-1 GOF mutation was considered due to moniliasis, onychomycosis, arthritis, hypergammaglobulinemia, and consanguineous marriage. In targeted next-generation sequencing (TNGS), A homozygous mutation in the AIRE gene SAND domain (c.769C > T, p.Arg257Ter) was detected (Figure 1). Then, the patient was diagnosed with APECED syndrome. The parents were heterozygous for the same mutation.

After a short while, he had cough and fatigue and was hospitalized with the diagnosis of pneumonia. In the lung examination, diffuse crepitant rales were present, more prominent on the left. Acute phase reactants were found to be high. Community-acquired pneumonia was considered, with no sequelae. Coronavirus 229E/NL6 was detected in the respiratory virus panel. IVIG treatment was given to prevent chronic infections and for immunomodulation purposes, as it is a Th17-mediated disorder, and T cell assistance was not sufficient.

Dental caries developed in the follow-up of our patient. No other clinical problems were encountered in the next two years with IVIG treatment and fluconazole and trimethoprim sulfamethoxazole prophylactic treatment. He also recovered from arthritis very well without any other medication. At the age of five, hypoparathyroidism developed with low calcium, high phosphorus, low parathormone, and high 25OHD vitamin levels. He is still on treatment with calcium lactate, calcitriol, trimethoprim sulfamethoxazole, fluconazole prophylaxis, and IVIG, and he is doing well.

## 3. Discussion

The *AIRE* gene causing APECED syndrome was first identified in 1997 [3]. This gene encodes transcriptional regulators primarily expressed in medullary thymic epithelial cells and contributes to the development of central immune tolerance in the thymus [5]. APECED is typically characterized by autosomal recessive inheritance, but autosomal dominant inheritance has also been reported. Compound heterozygosity is also common [1]. More than 126 AIRE mutations have been identified up to date. They are varying from single-nucleotide substitutions to large deletions spreading out across the coding sequence [3].

A cluster of missense mutations was detected in the CARD and PHD1 domains [6]. The APECED protein contains two zinc fingers of the plant homeodomain type (PHD), a DNA-binding domain, dubbed “SAND” [7]. The most common worldwide mutation is the FIN major mutation in the SAND domain in p.r257. It is dominant not only in Finland but also in many Eastern European countries. The SAND domain is a conserved 80 amino acid sequence likely to mediate protein-protein interaction and DNA binding. A number of nonsense mutations were located in this domain [3]. Our patient also had a homozygous mutation in the SAND domain which was published before [8].

AIRE is a transcriptional regulator primarily expressed in medullary thymic epithelial cells (mTECs) and plays an important role in thymocyte training and negative selection by controlling the expression of peripheral antigens in the thymus [9]. AIRE deficiency in the thymus impairs presentation of self-antigens that inhibit negative selection of autoreactive T cells [10]. In the absence of AIRE expression, autoreactive T cells undergo clonal deletion or transform into regulatory T cells, rushing to the periphery where they cause autoimmune destruction [5]. Faulty function in the AIRE gene may impair the clearance of autoreactive T cells and the development of Treg cells in the thymus. Patients with APECED show low expression of forkhead box protein P3 (FOXP3), which is critical for Treg cell activation and proliferation [10]. They are key mediators in peripheral tolerance and prevention of autoimmunity [4]. Our patient developed autoimmune manifestations such as hypoparathyroidism and arthritis.

Enamel hypoplasia, gastrointestinal dysfunction, and urticarial eruption may also occur [5]. Abnormalities such as ectodermal nail dystrophy and tympanic membrane calcification have been reported [1]. Our patient also developed dental problems, urticarial rash, and nail dystrophy.

The disease may also present with pneumonia and autoimmune hepatitis [11]. Our presented case had a severe pneumonia before IVIG therapy. The clinical spectrum of APECED may be highly variable even among siblings with the same AIRE genotype [3]. The AIRE protein is found not only in immunologically important tissues such as thymic medullary epithelial cells but also in peripheral blood, monocytes, and dendritic cells. This widely and different expression explains the variability of heterogenous symptoms in APECED patients [12].

Inflammatory arthritis, though very rare in APECED cases, may be an early manifestation [1]. Four patients with juvenile arthritis or misdiagnosed as JIA and lately diagnosed as APECED have been reported up to date (Table 1).

As shown in Table 1, novel compound heterozygous mutations were defined in a female case, diagnosed with APECED at the age of 10, and clinical findings of polyarticular JIA were observed at the age of 2 years (2nd patient). Carpopedal spasm and hypoparathyroidism were also observed in the follow-up of this patient [7]. Third case in Table 1 was a 2-year-old female patient who was diagnosed with systemic JIA with recurrent fever attacks, rash, and arthralgia. This case developed hypoparathyroidism at the age of 7 years, adrenal insufficiency at the age of 8 years, and ovarian insufficiency at the age of 12 years [12]. As in our patient, 4th case had dental problems that appeared in the early period and hypoparathyroidism developed at the age of five. After three years, she developed chronic mucocutaneous candidiasis, type 1 diabetes mellitus, adrenal insufficiency and ovarian insufficiency, and alopecia totalis [13]. Recurrent fever attacks accompanied by rash, enteritis, and swelling in the hand were observed in the 5th case. Then, she developed chronic mucocutaneous candidiasis, autoimmune hepatitis, nail mycosis, hypothyroidism, diarrhea, hypoparathyroidism, subclinical adrenal insufficiency, candida esophagitis, and vitiligo [14]. Identified cases are female, but our case is male.

A single-nucleotide polymorphism (SNP) that was strongly associated with APECED was identified in a large-scale genome-wide cohort of patients with rheumatoid arthritis. Two SNPs in AIRE gene, rs2075876 and rs760426, showed strong association with rheumatoid arthritis risk suggesting that in addition to the key AIRE gene mutations and other genetic and environmental factors, SNPs may have an impact on the phenotypic expression of APECED [2]. In addition, it is important to emphasize that a delay in diagnosis in a patient with arthritis may lead to poor prognostic outcomes.

In conclusion, inflammatory arthritis is rarely described in association with APECED and is misdiagnosed as juvenile idiopathic arthritis. In APECED cases, nonclassical symptoms such as arthritis may occur before developing classical symptoms and considering the diagnosis of APECED in patients with CMC and arthritis is very important for early diagnosis before development of complications and management of disease.

## Figures and Tables

**Table 1:** APECED cases presenting with arthritis and diagnosed as juvenile idiopathic arthritis on admission and other clinical features and AIRE genotype in the literature.

Gender	First manifestation/age of onset (years)	Later manifestations	Age of diagnosis (years)	AIRE genotype mutation	Nationality	Reference no.
(1) Male	Arthritis/3 y	Nail dystrophy, onychomycosis, pneumonia, dental caries, hypoparathyroidism	4	Homozygous c.769C > T, p.Arg257Ter	Turkish	Our case
(2) Female	Juvenile idiopathic arthritis (JIA)/2 y	Carpopedal spasms, hypoparathyroidism	10	Compound heterozygosity for c.232T > A and c.64_69del (p.V22_D23del)	Italian	[7]
(3) Female	Systemic JIA/2 y	Asthma-like dyspnea, hypoparathyroidism, adrenal failure, ovarian failure, chronic otitis media, pernicious anemia	17	c.892G > A (p.Glu298LYs)	Serbian	[12]
(4) Female	Juvenile idiopathic arthritis (JIA)/3 y	Tetralogy of Fallot, hypoparathyroidism, chronic mucocutaneous candidiasis, type 1 diabetes, adrenal insufficiency, growth hormone deficiency, ovarian failure, alopecia totalis, pernicious anemia	26	Declined testing for AIRE gene	Canadian	[13]
(5) Female	Systemic JIA at the age of one year with recurrent episodes of fever, rash, enteritis, hand swelling/1 y	Chronic mucocutaneous candidiasis, autoimmune hepatitis, nail mycosis, hypothyroidism, hypoparathyroidism, subclinical adrenal insufficiency, candida esophagitis, vitiligo	5	Homozygous mutation c.462G > A	German	[14]

## Data Availability

The data used to support the findings of this study are available from the corresponding author upon request.

## Abbreviations

APECED: Autoimmune polyendocrinopathy-candidiasis-ectodermal dystrophy
APS-1: Autoimmune polyglandular syndrome type 1
AIRE: Autoimmune regulatory gene
CMC: Chronic mucocutaneous candidiasis
CRP: C-reactive protein
ESR: Erythrocyte sedimentation rate
SAA: Serum amyloid A
ANA: Antinuclear antibody
Anti-ds DNA: Anti-double-stranded DNA antibody
ASO: Antistreptolysin O
RF: Rheumatoid factor
PPD: Purified protein derivative
NSAIDs: Nonsteroidal anti-inflammatory drugs
STAT-1 GOF: Signal transducer and activator of transcription 1 gain-of-function
TNGS: Targeted next-generation sequencing
IVIG: Intravenous immunoglobulin
PHD: Plant homeodomain type
FOXP3: Forkhead box protein P3
SNP: Single-nucleotide polymorphism.
